# The PREHAAAB Trial: Multimodal prehabilitation for patients awaiting open abdominal aortic aneurysm repair – A study protocol for an international randomized controlled trial

**DOI:** 10.1371/journal.pone.0339473

**Published:** 2025-12-29

**Authors:** Miquel Coca-Martinez, Robert J. Doonan, Chelsia Gillis, Kent Mackenzie, Daniel I. Obrand, Jason P. Bayne, Oren K. Steinmetz, Elie Girsowicz, Manuel Lopez-Baamonde, Samuel Jessula, Xavier Yugueros, Graciela Martinez-Palli, Heather L. Gill

**Affiliations:** 1 Department of Anesthesiology and Intensive Care, Hospital Clinic, Universitat de Barcelona, Barcelona, Spain; 2 Department of Anesthesiology, Hôpital Maisonneuve-Rosemont, Université de Montréal, Montreal, Canada; 3 Division of Vascular Surgery, Department of Surgery, McGill University, Montreal, Canada; 4 School of Human Nutrition, McGill University, Montreal, Canada; 5 Department of Vascular Surgery, Jewish General Hospital, Montreal, Canada; 6 Division of Vascular Surgery, Department of Surgery, Dalhousie University, Halifax, Canada; 7 Department of Vascular Surgery, Hospital Clinic de Barcelona, Universitat de Barcelona, Barcelona, Spain; PLOS: Public Library of Science, UNITED KINGDOM OF GREAT BRITAIN AND NORTHERN IRELAND

## Abstract

**Background:**

Patients undergoing open abdominal aortic aneurysm (AAA) repair remain at high risk for postoperative complications, particularly when presenting with limited baseline functional capacity. Prehabilitation, encompassing physical, nutritional, and psychological optimization before surgery, may improve outcomes in this population. However, evidence is limited in the context of open AAA repair.

**Objective:**

To evaluate the effectiveness of a multimodal prehabilitation program in reducing the incidence of postoperative complications in patients undergoing open AAA repair.

**Methods:**

This is a multicenter, international, randomized controlled trial. Eligible participants include adults scheduled for elective open AAA surgery. A total of 152 participants will be randomized 1:1 to receive either standard preoperative care or a 6-week multimodal prehabilitation program consisting of supervised exercise training, individualized nutritional support, and psychological counseling. The primary outcome is the rate of postoperative complications as measured by the Comprehensive Complication Index (CCI). Secondary outcomes include length of hospital stay, and readmission rates, changes in functional capacity, health-related quality of life and anxiety & depression. Data will be analyzed on an intention-to-treat basis. The study complies with SPIRIT 2013 guidelines and is registered at ClinicalTrials.gov (NCT05756283).

**Discussion:**

This trial will address a significant evidence gap regarding preoperative optimization in open abdominal aneurysm surgery. Findings will inform best practices for reducing morbidity in this high-risk population and contribute to enhanced recovery strategies in vascular surgery.

**Trial registration:**

[NCT05756283]

## 1. Introduction

### 1.1 Background and rationale

It is estimated that 20000 Canadians are diagnosed yearly with an abdominal aortic aneurysm (AAA) [1]. AAAs are usually asymptomatic until they rupture, which carries an 80–90% overall mortality [2,3]. In order to prevent rupture, AAAs are repaired when they reach a diameter of 5.5 cm in men and 5.0 cm in women [4]. Patients with AAA are highly comorbid, often have poor functional status and are considered high risk for adverse surgical outcomes [5]. AAAs can be repaired either by endovascular repair (EVAR) or open surgical repair. While EVAR may be less invasive, approximately 30–50% of patients are not candidates due to anatomical considerations [6]. In addition, it results in increased reinterventions and may have worse long-term survival [7–14]. Therefore, open repair remains a viable intervention in many patients; unfortunately, it is associated with a 3.6–4.2% mortality, and a morbidity of 30–40% [15–17]. Despite improvements in perioperative care, the rate of complications in open repair has remained relatively stable for the past 10–15 years [16]. In the short term, post-operative complications lead to an increased likelihood of dying in hospital, prolonged hospital stays, slower return to baseline, increased readmission rates, and decreased quality of life. In the long term, they can lead to decreased life expectancy, undermine physical and mental health, decrease patients’ functional status, and possibly threaten their independence [18–20]. Not only do complications affect the patients and their caregivers, but they also result in significant economic burden to the health care system and society as a whole [18–20].

Functional status is an individual’s ability to perform the normal daily activities required to meet basic needs, fulfill usual roles, and maintain health and well-being [21]. It has been shown to be a true measure of perioperative risk, and when functional status is poor, it is associated with worse perioperative outcomes [22–24]. The risk factors leading to poor functional status such as smoking and unhealthy behaviors are common in patients with AAA [25]. As such, individuals with AAA have significantly lower functional status and more comorbidities than aged-matched controls [26]. Fortunately, functional status is a modifiable preoperative risk factor that, if improved before surgery, leads to a decrease in postoperative morbidity [27,28]. The use of the preoperative time to optimize functional status to the goal of improving surgical recovery and perioperative morbidity is called prehabilitation [29–33]. Multimodal prehabilitation (MP) targets the three main pillars of functional status, namely, physical, nutritional, and psychological well-being [34]. Physical exercise has been the main component of most unimodal and multimodal prehabilitation programs with all types proving beneficial, from resistance training, to low, moderate, and high intensity interval training (HIIT) [35–38]. Nutritional status, the second component of MP, is independently associated with postoperative morbidity and mortality [39], and supports optimal gains in exercise-induced functional capacity [40]. Malnourishment exacerbates the catabolic state induced by surgery, limiting healing and surgical recovery [41]. Nutritional interventions are more effective in maintaining lean body mass if instituted before surgery than if commenced after [42,43]. Finally, psychosocial wellbeing and mental health, often overlooked, are an important component of recovery. Undergoing surgery is linked to emotional stress. Several studies have demonstrated the association between anxiety and depression with adverse postoperative outcomes, including increased length of hospital stay, infection, and poor wound healing and increased mortality [44–47]. MP addresses all three elements in one intervention because while important individually, they create a synergistic effect when combined [48]. Several studies have demonstrated that prehabilitation improves functional recovery and facilitates the recovery of daily activities of cancer participants after major surgery [23,49]. Improvements are greater in patients with poor baseline physical fitness, with prehabilitation decreasing overall complications and their severity after major surgery [50]. In high-risk patients undergoing major abdominal oncological surgery, prehabilitation decreased complications by 50% [28]. While there is high-quality evidence that backs up the feasibility and safety of HIIT programs on patients scheduled to undergo elective AAA repair, the evidence supporting prehabilitation’s capacity to reduce complications in AAA surgery is insufficient and heterogenous with no trial using the multimodal approach and most of the trials mixing cohorts of open and endovascular repair [26,51–53]. It seems logical to think that patients undergoing open AAA repair would benefit more from MP as this group clusters a higher incidence of postoperative complications compared to the endovascular repair patients. In conclusion, there is a lack of and a need for, high quality RCTs on multimodal prehabilitation before open AAA repair.

## 2. Objectives

Primary Objective: To investigate the role of multimodal prehabilitation in decreasing postoperative complications measured by the Comprehensive Complication Index (CCI) in patients undergoing open AAA repair.

Secondary Objective(s): To investigate the effects of multimodal prehabilitation on perioperative functional capacity, Health-Related Quality of Life, anxiety and depression, intensive care unit (ICU) stay, length of hospital stay, readmission rates, emergency visits, and cost-effectiveness of such program. The trial will also assess the compliance, fidelity, participant satisfaction and safety of the intervention.

## 3. Methods and analysis

### 3.1 Trial design and ethics

The trial protocol was written following the Standard Protocol Items: Recommendations for Interventional Trials (SPIRIT) guidelines [54]. This is a prospective, international, multicenter, open label, randomized controlled trial with an allocation ratio 1:1 comparing standard of care versus 6 weeks of a multimodal prehabilitation program. The recruitment period started in September 2024 and will last until September 2027. Recruitment of participants will take approximately three years, the follow-up and data collection after recruitment phase will last one year, and results will be analysed within a year of finishing. Both primary endpoint and baseline data will be blindly assessed. Intention-to-treat criteria will be used to define the analysis population; all randomized participants will be included in the analysis according to assigned group. The trial is registered on the Registry of National Institutes of Health ClinicalTrials.gov, a database of privately and publicly funded clinical studies conducted around the world (NCT05756283). The trial will be conducted according to the rules of Good Clinical Practice. The study will be conducted according to the principles of the Declaration of Helsinki and in accordance with the Medical Research Involving Human Subjects Act (WMO) and has been reviewed by the ethics board of McGill University Health Centre MP-37-2023-8983. No datasets were generated or analysed during the current study. All relevant data from this study will be made available upon study completion.

### 3.2 Setting

This trial will be conducted in the following academic tertiary hospitals: McGill University Health Centre (Montreal, Canada), Sir Mortimer B. Davis Jewish General Hospital (Montreal, Canada), Hospital Clínic de Barcelona (Barcelona, Spain) and Dalhousie University affiliated Hospitals (Nova Scotia, Canada).

### 3.3 Eligibility criteria

#### 3.3.1 Inclusion criteria.

Participants older than 50 years of age awaiting elective open AAA repair with AAA diameter ≤6.9 cm. Participants must have the ability to give individual informed consent.

#### 3.3.2 Exclusion criteria.

- Participants undergoing thoracic, thoracoabdominal and/or perivisceral AAA repair.
- Participants with ruptured or symptomatic AAA.
- Participants with AAA maximal diameter ≥7 cm.
- Physical inability to exercise: severe osteoarthritis, musculoskeletal or neurological impairment that precludes exercise.
- Contraindication to exercise: rest systolic blood pressure ≥ 180 mmHg and/or diastolic ≥ 100 mmHg, uncontrolled atrial or ventricular arrythmias or proven exercise induced arrhythmias, unstable angina, unstable or acute heart failure, severe symptomatic valvular stenosis, dynamic left ventricular outflow tract obstruction or other comorbidities that imply clinical instability.
- Cognitive impairment that would impede understanding of study procedures, informed consent or study questionnaires or the inability to effectively communicate in English/French/Spanish/Catalan.

### 3.4 Study outcomes

#### 3.4.1 Primary outcome.

The primary outcome of this study is the incidence of postoperative complications measured by the Comprehensive Complication Index. The CCI was developed to address some of the limitations of traditional surgical morbidity reporting, such as only reporting the most severe complication or only picking the most common types of complications [55,56]. It provides a continuous score from 0 (no complications) to 100 (death) that integrates the cumulative burden of all complications and their severity. It has been validated in surgical abdominal, urologic, and cardiothoracic patient populations [47,57,58]. It correlates better than any other scale with respect to length of stay, cost/burden to the health care system, readmissions, and disability [59–61].

#### 3.4.2 Secondary outcomes.

Secondary outcome measures will fall into 5 categories:

- Healthcare-related: Intraoperative complications, ICU length of stay, hospital length of stay, reintervention, 6-week and 1-year mortality/ readmission/ emergency room visit and prehabilitation cost-effectiveness.
- Change in functional and aerobic capacity measured by:
  - 6-minute walk test: the 6MWT evaluates the ability of an individual to maintain a moderate level of physical activity reflective of the activities of daily living [62]. As daily activity is mostly pursued at a submaximal level, functional walking capacity is a more direct measure of capacity for daily routine. Therefore, it is a useful tool to assess postoperative recovery and return to normal life [63].
  - VO2-peak, VO2-AT, VE/VCO2-AT: Cardiopulmonary Exercise Testing (CPET) is the current gold standard to assess aerobic capacity [64]. It provides an assessment of the integrative exercise responses involving the pulmonary, cardiovascular, hematopoietic, and skeletal muscle systems [64]. VO2 peak, VO2-AT and VE/VCO2-AT are CPET derived variables that predict postoperative morbidity and mortality, as well as indicators of cardiorespiratory fitness.
  - Yale physical activity survey (YPAS): YPAS is a self-reported measure of physical activity, comprising 18 activities evaluated according to the total number of hours done during an average week [65].
- Patient reported outcomes:
  - Health-related quality of life (SF-36): the SF-36 is the most widely used health-related quality of life measure and has been validated for surgical populations. It incorporates behavioral functioning, subjective wellbeing, and perceptions of health. Two summary scores have been developed: the Physical Component Summary and the Mental Component Summary, standardized to have a mean of 50 and a standard deviation of 10. A higher score on the SF-36 sub-scales or component summary measures indicates a better quality of life. A change of as little as 2 units on the physical component summary has been shown to be the minimum clinically meaningful change; 5 points is often targeted by medical intervention studies, though surgical interventions can have an impact as large as 10 points [66,67]. Data obtained from the SF-36 will be used to calculate the quality adjusted life years using the SF-6D [66].
  - Hospital Anxiety and Depression Scale (HADS): HADS is a reliable instrument for screening clinically significant anxiety and depressive symptoms in medically ill patients [68]. It has also been shown to be a valid measure of their severity [68]. It includes items that assess generalized depression and anxiety, including tension, worry, fear, panic, difficulties in relaxing, and restlessness.
- Nutritional status outcomes
  - Patient Generated Subjective Global Assessment (PGSGA): the PGSGA is a screening tool used to identify malnutrition and consists of two parts. The first part is the Patient Generated Subjective Global Assessment Short Form which is a widely validated malnutrition screening tool and has been previously used in vascular surgery [69–71]. The second part is completed by a dietitian and includes a physical examination, where fat and muscle stores and fluid status are evaluated, and an assessment of the disease/condition. The global score generates a subjective category rating for nutritional status.
  - Body composition: Weight, BMI, Fat free mass (kg) and body fat mass (%) will be assessed using bioimpedance.
  - Diet: A registered dietitian will assess usual energy and protein intake, including supplements, at baseline and pre-surgery using three-day food records or 24-hour dietary recalls.
- Study related outcomes:
  - Compliance: compliance to supervised and home-based exercise will be calculated by counting the number of sessions attended by the participant. Home-based exercise compliance will be estimated by counting the number of sessions they logged in an exercise logbook. Compliance to nutrition will be evaluated based on the assessment of dietary changes/goals set by the dietitian. Every week participants will be asked if the nutrition goals set have been met.
  - Fidelity: fidelity to supervised and home-based exercise will be recorded. Supervised exercise fidelity will be measured by recording the watts (for endurance training) and kg (for strength training) at which the participants train during the session (versus expected according to the individualized plan) as well as heart rate and RPE for both breathing and muscular fatigue [72].
  - Satisfaction and acceptability of the intervention: a satisfaction survey enquiring about the different components of the prehabilitation will be given upon completion of the intervention (S1 File).
  - Safety: safety will be assessed by recording the appearance of any adverse event occurring during assessments or intervention. A list of adverse events can be found in S5 File.

### 3.5 Study outline and procedures

The study CONSORT diagram, an overview of the study flow and the measurements taken during the PREHAAAB study can be found in .

**Fig 1:**
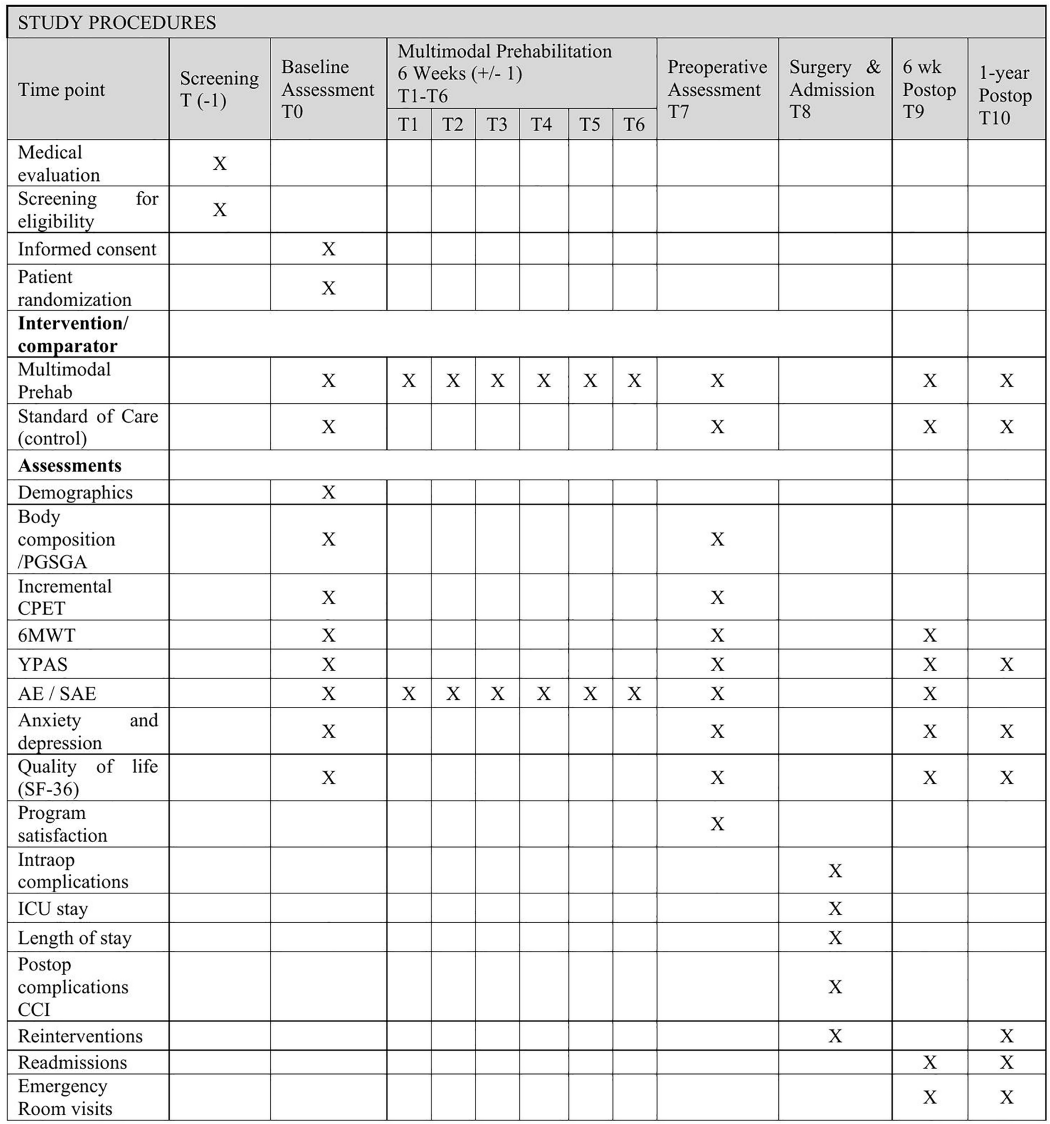
SPIRIT participant timeline; Schedule of enrollment, interventions, and assessments. PGSGA: Patient-Generated Subjective Global Assessment; CPET Cardiopulmonary Exercise Testing; 6MWT 6-Minute Walk Test; YPAS Yale Physical Activity Survey; AE Adverse Event; SAE Serious Adverse Event; CCI Comprehensive Complication Index.

**Fig 2:**
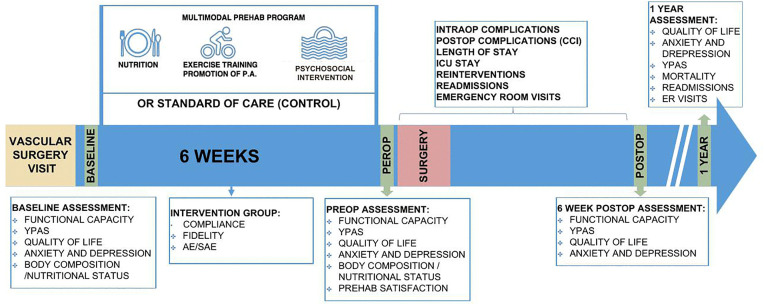
Study CONSORT diagram. AAA Abdominal Aortic Aneurysm; CPET Cardiopulmonary Exercise Testing.

**Fig 3:**
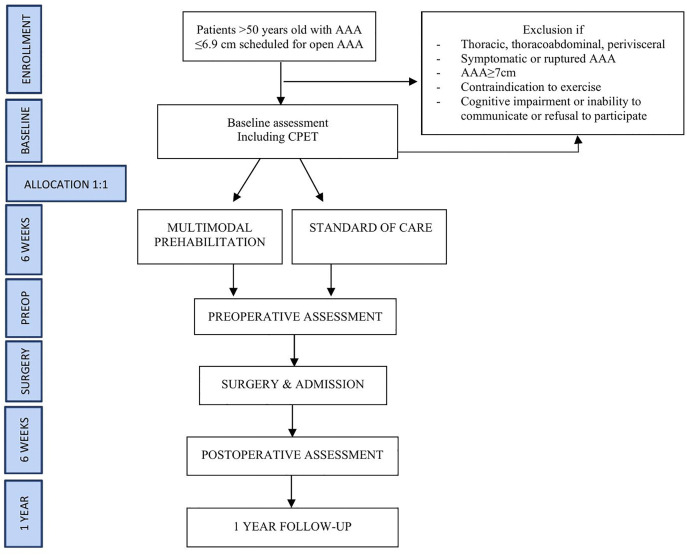
Study flow. YPAS Yale Physical Activity Survey; AE Adverse Event; SAE Serious Adverse Event; CCI Comprehensive Complication Index; ER Emergency Room.

#### 3.5.1 Participant screening, recruitment and informed consent (T-1).

In all participating centers, participant selection will begin after a high probability of having open AAA repair is stablished by the perioperative vascular team. Within one week of the decision to go for surgery, a medical research team will screen for health conditions that preclude study participation. The participant will be contacted by a study coordinator who will ask for willingness to participate in the study. The participant will be offered the possibility to participate in the trial and, if the participant agrees, a first baseline appointment (T0) will be scheduled where written and oral information about the trial will be given.

The study coordinator will answer any questions that participants might have and ask if the patient is willing to participate in the trial. The right of a patient to refuse participation without giving reasons will be respected. The patient will remain free to withdraw at any time from the study without consequences for further treatment.

#### 3.5.2 Informed consent, baseline assessment and randomization (T0).

Before starting the baseline assessment, participants will be given enough time to request more trial details and to decide whether they wish to participate. Participants will be required to sign the informed consent form.

After signing the informed consent all participants will undergo a full blinded baseline assessment that consist of the following:

- Past medical history, including characteristics of AAA (diameter and location), current medication, ASA status [73], Charlson Comorbidity Index [74], and CSHA Clinical Frailty Scale [75].
- Resting blood pressure, heart rate and oxygen saturation.
- Participant demographics: age, sex, gender, and education level.
- Body composition: height, weight, body mass index, Fat-Free Mass (kg), fat mass. Nutritional status will be assessed using the Patient Generated Subjective Global Assessment (PG-SGA).
- 6-Minute Walk Test, which will assess functional capacity in relation to activities of daily living). The 6MWT will be executed following the American Thoracic Society guidelines for the 6MWT [62].
- Cardiopulmonary exercise test (CPET) conducted by an exercise physiologist/kinesiologist/physiotherapist with expertise in comorbid population under the supervision of a physician. Reliability and safety of CPET in participants with AAA have been previously demonstrated [26,36,51]. CPET will be performed following the iPOETSS - International Prehabilitation and Exercise Testing and Training Society guidelines in all centers [76]. The data obtained during CPET (physiological variables in response to exercise and workload in watts at the anaerobic threshold and at peak/maximum exercise) will be used in the intervention group to tailor the intensity at which the participants will perform the aerobic exercise prescription. If a non-previously diagnosed pathology that poses a threat to the health of the participant (such as an exercise contraindications as listed in the exclusion criteria) is detected with the CPET, the patient will be excluded from the study and will be referred to the appropriate health care resource.
- Physical activity measured with the Yale Physical Activity Survey (YPAS).
- Health-Related Quality of life using the Medical Outcomes Study SF-36 V.2 questionnaire. This quality-of-life questionnaires has been previously used in this population.
- Anxiety and Depression measured using the Hospital and Anxiety scale (HADS).

***Randomization*:** Randomization will be performed after baseline assessment. Participants will be randomized to both arms equally a (1:1 ratio) upon final confirmation of eligibility. A stratified randomization will be performed by site and sex; with block size 2,4 and 6 to ensure unbiased allocation of the patients. Allocation to study group will be revealed using RedCap software to the research team that will carry out the intervention once baseline assessment is completed.

#### 3.5.3 Standard of care (T1-T6).

Participants in the control group will receive the standard of care according to each center’s practice. This will include comorbidity optimization with special emphasis on blood pressure management according to international hypertension guidelines, anemia correction and smoking cessation advice if deemed appropriate [77,78].

#### 3.5.4 Multimodal Prehabilitation program (MP) (T1-T6).

Participants in the intervention group will undergo, in addition to the standard of care, 6 ± 1 weeks of a personalized multimodal prehabilitation program. The interventions included will be patient-centered, aiming to optimize patients’ preoperative health status while enhancing their empowerment and engagement. The main components of the program will consist in:

**i. Supervised exercise program**: This will entail two 1-hour supervised sessions per week of aerobic and strength training, with at least one day of rest in-between. During the first session, an exercise specialist (kinesiologist, sport physician or physiotherapist) will assess participants’ mobility and capacity to undertake exercise. As previously mentioned, the exercise intensity for the endurance training will be defined and personalized based on the individual CPET values obtained during baseline assessment. The exercise specialist will also assess the maximum tolerated weight for each big muscle group using the eight-repetition maximum test that will allow to establish the initial weight for the strength training. Based on the evaluation, a personalized program from the protocolized program will be generated, as defined in S2 File. The supervised session will be carried out in a supervised setting (hospital or community-based facility). Heart rate, blood pressure, oxygen saturation (if lung disease present) and perceived exertion rate using the modified Borg scale (for both breathing and muscle fatigue) will be monitored throughout the training session. After each session of supervised exercise participant will receive 0.4g·kg of whey protein supplementation to promote anabolism unless contraindicated due to milk allergy or galactosemia in which case a soy-based protein will be provided [79].
  - **Supervised aerobic training** will consist of ~45 min of HIIT on a stationary bicycle. Each aerobic session will typically include 10 minutes of warm-up and 5 minutes of cool-down pedaling at 30%–40% of their peak workload attained during CPET. An interval training will consist in at least 5 bouts combining 2 min of high-intensity exercise. The high intensity bouts will start at 70% of peak workload and will be progressed in 5% weekly increases up to 90%–100%, although progression will be tailored on individual basis to ensure tolerance, safety and maximize the training. The high intensity bouts will be interspersed with 3 min recovery periods at lower intensity (40%–50% of the peak workload). Blood pressure, heart rate, SpO2, leg fatigue Rate of Perceived Effort (RPE-L) and breathing RPE (RPE-B) will be monitored throughout the session during high intensity bouts and during the recovery periods. A stepwise reduction of workload by 10% will be deemed pertinent if participant cannot tolerate the workload during peaks or if systolic blood pressure rises >180 mmHg or HR rises >95% of maximal HR attained during CPET. If HIIT is not well tolerated by the participant despite the progressive reduction of workload (repeated BP > 180 mmHg/ HR > 95% or other exercise intolerance during high intensity), an alternative exercise prescription will be proposed, consisting in moderate intensity continuous exercise (40–60% of peak workload or RPE 4–6/10).
  - **Supervised strength training** will consist of 2–3 upper (pectoralis and latissimus dorsi) and lower (quadriceps) limb exercises based on 2–3 sets of 8–12 repetitions always avoiding Valsalva’s maneuvers. The initial weight will be based on 60–70% of the eight-repetition maximum test. The weight and/or the number of repetitions will increase every week, if tolerated by the participant, to ensure strength progression and maximize the training effect. The weight and/or the number of repetitions will be decreased if systolic blood pressure rises >180mmHg or HR rises >95% of maximal HR.
**ii. Home-based training and promotion of physical activity**:

During the first session, the exercise physiologist/kinesiologist/physiotherapist will use motivational interviewing techniques to promote physical activity and healthy lifestyle. The motivational interview will focus on empathy, reflective listening, and affirmation, and will address the participants’ barriers and limitations related to physical activity. The role and safety of exercise and physical activity during the preoperative period will be discussed and participants will be taught on how to perform exercise on their own within the safety limits. Participants will follow a home-based exercise program containing aerobic, strength, and inspiratory muscle training (IMT) exercises. Participants will receive a logbook where the exercise specialist will prescribe the intensity, type, time, and frequency of aerobic, strength and IMT exercises. Each time the participant performs the prescribed home-based exercises they will register their activity in the logbook provided, including the RPE at which they trained. The exercise specialist will review the logbook every week and will adjust the home-based exercise plan accordingly to ensure both safety and exercise progression. Furthermore, all participants will receive a pedometer at the beginning of the 6-week period that will allow tracking of daily physical activity in both groups.

- **Aerobic exercises:** participants will be instructed on how to conduct aerobic exercises at home in a low-moderate intensity, by either walking or cycling initially at RPE 2–4/10 for 20 min at least once per week. This will be increased in stepwise increments by 10% each week if tolerated.
- **Inspiratory muscle training:** daily IMT with an inspiratory threshold-loading device (threshold IMT) will be prescribed. During the first visit with the exercise specialist the maximal inspiratory mouth pressure will be measured, and participants will be taught to perform daily IMT exercises at 30% of their maximal inspiratory mouth pressure. The pressure will be increased by 5% each week if the rate of perceived exertion scored on the Borg scale is < 5/10.
- **Strength exercises:** participants will be instructed to conduct low intensity whole-body muscular strength exercises at home using an elastic band at least one time per week. The exercises will be adapted to each individual’s capacity and could include 2–3 sets of 8 repetitions of wall push-ups, sit-ups, calf raises, hamstring kickbacks, or standing strides (lunges). Progression of the exercises will be made by increasing the number of repetitions up to 12, or by changing the exercise if deemed appropriate.

**iii. Nutritional assessment, education, and supplementation**

To enhance the anabolic effect of physical training, improve fat free mass and obtain or maintain an optimal nutritional status during the preoperative period, sufficient intake of energy nutrients (especially protein) is required [80]. Participants will meet with a registered dietitian for one initial session and at least one follow-up session. This session will include the following:

- **Assessment:** the dietician will assess the participant’s nutritional status using the parameters measured during baseline assessment (PG-SGA, body weight, height, Fat free mass, and body fat). Moreover, the participant will be asked to fill in a three-day food diary or conduct a 24h-recall prior to their meeting with the dietician, which will be used to estimate habitual macronutrient consumption.
- **Education:** learning correct portion sizes, improving diet quality by building a balanced plate and reducing ultra-processed foods, adequate fiber intake through whole grains, vegetables and legumes, learning sources and importance of protein, importance of timing and spacing of meals, encouraging food sources of amoga-3 fatty acids, along with practicing mindful eating. Promotion and encouragement of dietary changes to ensure balanced intake with adequate protein, weight management, and the regulation of blood glucose will be discussed. Possible barriers to attain the proposed dietary changes and ways to overcome them will also be discussed along with the importance of perioperative nutrition, using a COM-B questionnaire. Based on estimated habitual protein intake, participants will receive a tailored dietary advice aiming to meet a total protein intake of 1,2–1,5g/kg daily (ideal body weight if BMI > 30), unless contraindicated and 20–30 Kcal/kg daily (20 Kcal/kg for weight loss, 25 Kcal/kg for maintenance and 30 Kcal/kg for gain). [80]. Participants will be instructed to evenly ‘spread’ their protein intake over 3 meals with a minimum of 25–30 gram of protein/ meal.
- **Supplementation:** To stimulate muscle protein synthesis, participants will also be provided with 0,4g/kg (adjusted to ideal weight if BMI > 24.9) of whey protein powder immediately after supervised strength training (2 times a week). Micronutrient deficiencies, including vitamin D will be assessed by the dietician, who will recommend their supplementation as needed.

**iv. Psychosocial intervention**

It is expected that patients undergoing surgery feel some degree of anxiety and depression. Since both anxiety and depression can influence the motivation to carry out social and functional activities as well as partaking in the prehabilitation program, psychological strategies can be put in place to help patients to cope with the stress of surgery and disease. Therefore, participants will receive one hourly session of psychological intervention called “surgical stress tolerance training”. In this session the psychosocial specialist will address the patient’s anxieties, coping strategies, and post-operative expectations, with the goal of optimizing psychological well-being & ways of coping with surgery. The importance of the patient’s active participation in the preparation for surgery and healing process will also be discussed. All participants in the intervention group will be given instructions on relaxation and deep breathing techniques that they can practice at home with a guided relaxation audio that will be provided via CD or USB key. A follow-up session during the prehabilitation period will be offered to those participants who score high in anxiety and depression in the HADS questionnaire (8 points or more) or those participants who request an additional session.

#### 3.5.5 Preoperative assessment (T7).

Six weeks after the baseline assessment, or the week prior to surgery if surgery happens >6 weeks after baseline, participants will undergo a preoperative assessment with the same measurements performed at baseline assessment (resting blood pressure, heart rate oxygen saturation, body composition, PG-SGA, 6-Minute Walk Test, CPET, YPAS, SF-36, HADS questionnaire). Participants will be offered the possibility to continue training until their week of surgery; in case it gets delayed. In addition to these measurements, the intervention group will be invited to fill out a satisfaction survey regarding the different components of the prehabilitation group that can be found in the S1 File.

#### 3.5.6 Surgery and admission (T8).

Perioperative clinical protocols will be followed according to each site’s standard of practice independently of group allocation; [76]. Anesthesia procedures and surgery will be performed by the usual anesthesia and vascular surgery teams of each site. Hospital healthcare providers (nurses, anesthesiologist, vascular surgeons, physiotherapists, etc.) involved in the participants care will be blinded to the study group allocation, unless the participant discloses that information. To avoid that, participants will be advised not to disclose their allocation to healthcare providers. Any intraoperative deviation of the standard course or event will be recorded (S3 File). The use of blood products, as well as the need for high doses of vasopressors intraoperatively will also be recorded (high doses of one vasopressor or the use of two or more vasopressors) in the case report form. Postoperative care will not differ between groups and will follow the standard of practice for each site. Postoperative complications throughout admission will be assessed daily by a physician and will be recorded in the CRF by a research team member. Both team members will be blinded to the study group allocation to avoid bias. The primary outcome of the study, the Comprehensive Complication Index (CCI), will be calculated based on the number and severity of the complications recorded. A description of complications and their severity can be found in the S4 File. Length of ICU stay, need for a reintervention and hospital length of stay will be recorded.

#### 3.5.7 Postoperative assessment (T9).

Six weeks after discharge, participants will undergo a postoperative assessment where the following measurements will be performed:

- Resting blood pressure, heart rate and oxygen saturation.
- 6-Minute Walk Test
- Yale Physical Activity Survey
- SF-36 V.2 questionnaire
- HADs questionnaire

Emergency room visits and readmissions, from discharge to postoperative assessment, will also be assessed and recorded.

#### 3.5.8 1-year follow-up (T10).

One year after discharge participants will be contacted by a research team member via phone call to enquire about admissions to ER visits and hospital admissions. Hospital records will be also assessed to track any ER visits, hospital admissions and mortality. A package of questionnaires will be sent via postal mail or email which will include SF-36, HADs and YPAS.

### 3.6 Statistical analyses

#### 3.6.1 Sample size.

Sample size was calculated for the primary outcome of complications as defined by the CCI. From the literature, the mean CCI ranges from 25–35 after open AAA repair, and their standard deviations are 17–30. We have chosen 30 as the most clinically relevant mean difference for the CCI and standard deviation of 20. Given the inputs, we require a total of 126 patients to achieve standard 80% statistical power and 5% tolerance. After considering a 20% dropout rate, we require 152 patients (76 per group) in this study. Since one interim analysis was planned, a total of 40 patients were needed in each treatment arm at the time of analysis to adequately power the interim analysis based on the type-I error suggested by the O’Brien-Fleming method (a minimum P = 0.0054).

#### 3.6.2 Primary analysis.

The primary analysis will be conducted by the intent-to-treat approach; differences in CCI scores between MP and the standard-of-care arms will be assessed in 45 days. Descriptive statistics will be performed using means, proportions, medians, standard deviations, and interquartile ranges, as appropriate depending on the nature of the data. We will use Generalized Linear Mixed or Generalized Estimation Equation models to evaluate the impact of the intervention, accounting for between and within group differences. The effect of MP on specific postoperative complications will be analyzed using parametric tests for categorical variables. The effect of MP on functional improvement and quality of life will be analyzed using time interactions with the group after considering the site at a random level. In addition, we will perform the sensitivity analysis based on a per-protocol approach using mixed effects, with study site as the random effect to cross-validate our results. We also plan to perform subgroup analyses of the primary outcome measure, as well as MP adherence on the following groups: study site, sex, and other variables which have previously been shown to impact MP adherence as well as postoperative outcomes after AAA repair, such as age, diabetes, renal failure, and baseline functional status.

#### 3.6.3 Interim analysis.

An interim analysis will be conducted at the halfway (when we reach half the numbers) point of recruitment to assess the safety and the primary outcome endpoints. An independent statistician will perform the analyses, and the data safety monitoring committee will determine the results and decide about the trial’s continuation as designed.

#### 3.6.4 Cost-effectiveness analysis.

Medical costs will be calculated using a micro-costing approach with overhead costs allocated using the step-down method. Unit costs will be supplied from the hospital finance department or derived using provincial health ministry records. Healthcare resource utilization will be defined as the number of clinic visits for surgical, family medicine, and other visits. To capture episodes that do not occur within our healthcare network, a validated healthcare resource utilization questionnaire will be administered to patients at 45-days. The primary effectiveness measure of the economic evaluation will be utilities, which will be mapped and derived from the SF-36.

### 3.7 Risks benefits and exclusion of participants

Prehabilitation has been shown to be useful at reducing postoperative complications in high-risk patients. We expect that patients in the prehabilitation group will benefit from the intervention as it is intended to be a multimodal optimization tool prior to surgery. We expect them to improve their preoperative aerobic and functional capacity status, strength, nutritional status and to recover faster postoperatively with less postoperative complications. Based on previous literature of exercise in patients with AAA, we do not expect the presence of serious adverse exercise-related events. For instance, Weston et al. reported no serious adverse events in their HIIT trial in AAA patients, and only an event rate of 1.8 per 100 person-hours. (CI 0.4–9.6 events) for non-serious adverse events [81]. Nevertheless, a series of measures will be put in place to make sure that safety prevails throughout the study. Firstly, participants will be excluded if they present any contraindication to exercise. Furthermore, patients will undergo a cardiopulmonary exercise testing that will act both as a screening tool to uncover any non-diagnosed cardio-respiratory pathology and as safety tool by allowing us to determine the safe limits of exercise training. A referral to a specialist (cardiologist, pneumologist, internist) will be made if any event occurs during the assessments or training and the participant will be excluded from study participation.

## 4. Data collection, handling, and monitoring

De-identified data will be collected in different timepoints (Fig 1). The de-identified data will be uploaded in an electronic CRF using Research Electronic Data Capture (REDCap). The REDCap server containing de-identified data will be located in the McGill University Health Centre Research Institute facility.

### 4.1 Data Monitoring Committee

A Data Monitoring Committee (DMC) will be established to ensure data quality and transparency. This committee will be responsible of training research staff on data management and electronic CRF. The committee will also be responsible of ensuring that all sites have all the necessary requirements, material an equipment to initiate the trial and will monitor study procedures, as well as adherence to the protocol throughout the study. Should any protocol deviation occur during the trial in any of the sites, it will be documented and reported to the data monitoring committee.

## 5. Data Safety Monitoring Board (DSMB)

A Data and Safety Monitoring Board (DSMB) will be created for this trial. It will be formed by an independent group of experts. Their responsibilities will be to review the protocol before the trial starts, review and assess the study data for participant safety, evaluate study conduct, milestones, progress, and efficacy. They will be responsible of making recommendations concerning the continuation, modification, or termination of the trial.

The different research personnel of all trial sites will be responsible of reporting to the principal investigator any exercise-related adverse events during the assessments or the MP period. The PI will immediately report severe adverse events to the Data Safety Monitoring Board and the sponsor (within 24h of the SAE incidence).

### 5.1 Premature termination of the study

The DSMB will decide to terminate the trial in case of significantly increased incidence of serious adverse events. Serious adverse events might include aneurysm rupture, cardiac arrhythmias, or other potential exercise-related events during CPET or exercise sessions (S5 File for full list of adverse events).

## 6. Dissemination

The main results of this trial will be disseminated in a peer-reviewed journal. All other study results arising from secondary outcomes could be published as other articles, abstracts, or presentations in medical conferences.

## Supporting information

S1 File Satisfaction questionnaire. (DOCX)

S2 File Standardized exercise component of Multimodal prehabilitation. (DOCX)

S3 File Intraoperative complications. (DOCX)

S4 File Postoperative complications. (DOCX)

S5 File Adverse events. (DOCX)

S1 Checklist (DOCX)
