# Supplementary material for: The PREHAAAB Trial: Multimodal prehabilitation for patients awaiting open abdominal aortic aneurysm repair – A study protocol for an international randomized controlled trial
Source: PLoS One. 2025 Dec 29;20(12):e0339473. doi: 10.1371/journal.pone.0339473 (PMC12747390; doi:10.1371/journal.pone.0339473)
Supplement: S1 File — (DOCX) [file pone.0339473.s001.docx]

**S1: Satisfaction questionnaire**

**1. How satisfied are you with the prehabilitation program? Please check the box that corresponds to your answer**

| **0** | **1** | **2** | **3** | **4** | **5** |
| --- | --- | --- | --- | --- | --- |
| Not satisfied |  |  |  |  | Very satisfied |

**2. For you personally, what was the most important reason to join the program?**

- - My surgeon recommended the program
  - This program could help me prepare for my surgery
  - I wanted to get in shape
  - I wanted to feel accompanied and taken care of during the preoperative period
  - All the above
  - I was not motivated to join the program
  - Other reason, please specify:

**3. How challenging did you find the program?**

| **0** | **1** | **2** | **3** | **4** | **5** |
| --- | --- | --- | --- | --- | --- |
| Not challenging |  |  |  |  | Very challenging |

**4. Which component did you like most of the program?**

- - Training (aerobic and/or strength)
  - Protein Supplements
  - Dietary advice
  - Stress tolerance training
  - None of the above

**5. Which component was the most challenging component for you?**

- - Training (aerobic and/or strength)
  - Protein Supplements
  - Dietary advice
  - Stress tolerance training
  - None of the above

**6. If relevant, which factor(s) contributed the most to not attending the supervised sessions?**

- - Finding time
  - Arranging transport / Arranging parking
  - Motivation
  - Tiredness
  - I felt ill
  - Other hospital appointments interfering
  - Other factors, please, specify:

**Let us know how we did in your own words. Would you change anything from the program?**
