# Supplementary material for: The PREHAAAB Trial: Multimodal prehabilitation for patients awaiting open abdominal aortic aneurysm repair – A study protocol for an international randomized controlled trial
Source: PLoS One. 2025 Dec 29;20(12):e0339473. doi: 10.1371/journal.pone.0339473 (PMC12747390; doi:10.1371/journal.pone.0339473)
Supplement: S2 File — (DOCX) [file pone.0339473.s002.docx]

**S2: Standardized exercise component of Multimodal prehabilitation**

- ***EXERCISE***

All participants will undergo a cardiopulmonary exercise test. The supervised exercise intensity will be defined by the values obtained during the baseline CPET.

- - **Supervised exercise** *2 x week*
    - HIIT stationary bicycle (~45min): 10 min warm-up at 30-40% peak work rate, followed by at least 5 bouts of 2 min moderate to high-intensity 70-90% interspersed with 3 min recovery periods at lower intensity (40%–50% of the peak work rate). Workload reduction by 10% if SBP >180 mmHg or HR >95% of maximal HR.

% Peak work rate (watts)

Time

30-40%

40-50%

70-90%

2min

3 min

10 min

5 min

- - - Strength exercise: 2–3 upper and lower limb exercises based on 2–3 sets of 8–12 repetitions avoiding Valsalva maneuvers. At the first session, the exercise specialist will obtain the tolerated weight for the 8-repetition maximum for each muscle group. Participants will train at 60-70% of their 8-repetition maximum.
  - **Home-based exercise** / *promotion of physical activity*

- Promotion of PA: motivational interviewing, promotion of physical activity and home-based exercise prescription will be given during the first exercise session and adjusted every week.

- Aerobic exercise: low-moderate intensity, by either walking or cycling initially at intensity of 2-4/10 RPE for 20 min at least once per week, to be increased in stepwise increments by 10% each week, if tolerated.

- IMT exercises: IMT at 30% of maximal inspiratory mouth pressure for 15 minutes daily. The pressure will be increased by 5% each week if the rate of perceived exertion scored on the Borg scale is <5/10.

- Resistance exercise: 1x/week using elastic bands, along with flexibility exercises. Home-based whole-body muscular resistance will be adapted to each individual’s capacity and could include 2-3 sets of 8 repetitions of wall push-ups, sit-ups, calf raises, hamstring kickbacks, and/or standing strides (lunges), increasing the number to reach 12 repetitions if tolerated.
