# Supplementary material for: The PREHAAAB Trial: Multimodal prehabilitation for patients awaiting open abdominal aortic aneurysm repair – A study protocol for an international randomized controlled trial
Source: PLoS One. 2025 Dec 29;20(12):e0339473. doi: 10.1371/journal.pone.0339473 (PMC12747390; doi:10.1371/journal.pone.0339473)
Supplement: S3 File — (DOCX) [file pone.0339473.s003.docx]

**S3: Intraoperative complications**

- Clinically significant hemorrhage: intraoperative bleeding requiring transfusion of packed red blood cells (PRBC) during surgery or within 24 hours after surgery
- Bowel injury: injury of the small or large bowel requiring intraoperative repair or additional resection.
- Urinary tract injury: injury of the ureter or bladder requiring intraoperative repair
- Vascular injury: unexpected injury of any vessel requiring intraoperative repair besides intended AAA surgical repair
- Any intraoperative injury of any organ or viscera
- Cardiac or respiratory complications: any cardiovascular (e.g., cardiac arrhythmia, myocardial infarction) or respiratory (e.g., pneumothorax) complication occurring during surgery.
- Severe reperfusion syndrome
- Dural puncture during epidural catheter placement,
- Aspiration of gastric content: intraoperative pulmonary aspiration of gastric content
