# Supplementary material for: The PREHAAAB Trial: Multimodal prehabilitation for patients awaiting open abdominal aortic aneurysm repair – A study protocol for an international randomized controlled trial
Source: PLoS One. 2025 Dec 29;20(12):e0339473. doi: 10.1371/journal.pone.0339473 (PMC12747390; doi:10.1371/journal.pone.0339473)
Supplement: S4 File — (DOCX) [file pone.0339473.s004.docx]

**S4: Postoperative complications and severity grading**

Adapted from *Standards for definitions and use of outcome measures for clinical effectiveness research in perioperative medicine: European Perioperative Clinical Outcome (EPCO) definitions A statement from the ESA-ESICM joint taskforce on perioperative outcome measures(61).*

- Acute Kidney Injury (AKI)
- Postoperative hemorrhage
- Anastomotic breakdown
- Bowel ischemia
- Paralytic ileus
- Cardiac arrest
- Cardiogenic pulmonary edema
- Deep vein thrombosis (DVT)
- Delirium
- Gastrointestinal bleed
- Infection, source uncertain
- Laboratory confirmed blood stream infection
- Myocardial infarction, angina
- Myocardial injury after non-cardiac surgery (MINS)
- New cardiac arrythmia
- Congestive heart failure
- Acute Respiratory Distress Syndrome (ARDS)
- Pneumonia / respiratory tract infection / aspiration pneumonitis
- Pneumothorax
- Atelectasis
- Pleural effusion
- Pulmonary embolism (PE)
- Stroke / TIA
- Surgical site infection (superficial /deep / organ / space)
- Urinary tract infection
- Anesthesia-related postoperative complications: Post-dural puncture headache, epidural hematoma or abscess.

**Clavien-Dindo complication grading (61):**

| **Grade** | **Definition** |
| --- | --- |
| Grade I | Any deviation from the normal postoperative course without the need for a pharmacological treatment or surgical, endoscopic, or radiological interventions. |
|  | Permitted therapeutic interventions include drugs such as anti-emetics, analgesics, diuretics, electrolytes, and physiotherapy. This grade also includes wound infections opened at bedside. |
| Grade II | Requiring pharmacological treatment with drugs other than such allowed for grade I. Blood transfusion and total parenteral nutrition are also included. |
| Grade III | Complication requiring surgical, endoscopic or radiological intervention |
| IIIa | Intervention not under general anesthesia |
| IIIb | Intervention under general anesthesia |
| Grade IV | Life-threatening complication (including central nervous system complications), requiring critical care. |
| IVa | Single organ disfunction |
| IVb | Multi-organ disfunction |
| Grade V | Death of the patient |
