# Supplementary material for: The PREHAAAB Trial: Multimodal prehabilitation for patients awaiting open abdominal aortic aneurysm repair – A study protocol for an international randomized controlled trial
Source: PLoS One. 2025 Dec 29;20(12):e0339473. doi: 10.1371/journal.pone.0339473 (PMC12747390; doi:10.1371/journal.pone.0339473)
Supplement: S5 File — (DOCX) [file pone.0339473.s005.docx]

**S5: Adverse Events**

| **System** | **Adverse Event** | **Severity** |
| --- | --- | --- |
| Cardiovascular | Angina or chest pain | Mild to severe |
|  | Arrhythmia | Mild to severe |
|  | Hypertension or hypotension episodes | Mild to severe |
|  | Syncope or presyncope | Moderate-Severe |
|  | Myocardial infarction | Severe |
|  | Sudden cardiac arrest | Severe |
| Respiratory | Extreme dyspnea disproportionate to effort | Mild to severe |
|  | Exercise-induced bronchospasm/asthma | Mild to severe |
|  | Hypoxemia or desaturation | Mild to severe |
|  | Hyperventilation / respiratory alkalosis | Mild |
| Musculoskeletal | Muscle strains, tears, or cramps | Mild |
|  | Tendon or ligament injuries | Mild to severe |
|  | Joint sprains or dislocations | Moderate to severe |
|  | Exacerbation of arthritis or pain | Mild to severe |
|  | Rhabdomyolysis | Mild to severe |
| Neurological | Dizziness or vertigo | Mild |
|  | Falls with trauma/fractures | Severe |
|  | Seizures | Severe |
|  | Stroke | Severe |
| Metabolic/Systemic | Hypoglycemia | Moderate to severe |
|  | Hyperglycemia | Mild to moderate |
|  | Electrolyte disturbances | Mild to moderate |
|  | Dehydration, heat exhaustion/heat stroke | Mild to moderate |
|  | Fatigue and overexertion | Mild |
| Gastrointestinal | Nausea/vomiting/indigestion/diarrhea | Mild |
|  | GERD exacerbation | Mild |
|  | Abdominal cramps | Mild |
| Psychological | Anxiety or panic attacks | Mild to moderate |
|  | Fear of cardiac event/loss of control | Mild |
